# A qualitative study exploring the clinical phenomenology and impact of hypersexuality in patients with Parkinson’s Disease

**DOI:** 10.1038/s41598-024-79966-z

**Published:** 2024-11-20

**Authors:** Natalie Tayim, Jalesh N. Panicker, Jennifer Foley, Caroline Selai, Walaa G. El Sheikh

**Affiliations:** 1 Doha Institute for Graduate Studies, Doha, Qatar; 2 Department of Uro-Neurology, The National Hospital for Neurology and Neurosurgery, Queen Square, UCLH NHS Foundation Trust, London, UK; 3 Department of Brain Repair and Rehabilitation, UCL Queen Square Institute of Neurology, Faculty of Brain Sciences, University College London, London, UK; 4 Department of Neuropsychology, National Hospital for Neurology and Neurosurgery, Queen Square, WC1N 3BG London, UK; 5 UCL Queen Square Insitute of Neurology, WC1N 3CG London, UK; 6 Department of Clinical and Movement Neuroscience, UCL Queen Square Institute of Neurology, Faculty of Brain Sciences, University College London, London, UK; 7 Faculty of Medicine, American University of Beirut, Beirut, Lebanon

**Keywords:** Hypersexuality, Parkinson’s Disease, Clinical phenomenology, Qualitative, Neuroscience, Psychology, Health care, Neurology

## Abstract

Hypersexuality (HS) is a complex yet underreported phenomenon in Parkinson’s disease (PD), profoundly impacting patients’ lives. This study aims to systematically investigate the clinical phenomenology and manifestations of HS using qualitative methodologies. This phenomenological qualitative research study employed semi-structured interviews to examine hypersexuality and its impact among nine English-speaking individuals with PD. Participants were recruited from clinical settings and met specific inclusion criteria, including clinically diagnosed PD, reported hypersexuality post-PD diagnosis, and no cognitive impairment. The study adhered to ethical standards, with written informed consent obtained from all participants. Nine patients with PD (six males, mean age 61.7 ± 13.3 years, and three females, mean age 64.3 ± 5.7 years) participated. The mean age of PD onset was 51.4 ± 12.5 years, while HS onset was 54.1 ± 11.5 years, ranging from 35 to 68 years. Eight of the nine patients were in monogamous relationships. Qualitative analysis revealed ten themes. Clinical manifestations included increased preoccupation with sex, heightened desire, and altered behaviors like risk-taking. Sexual practices varied, with increased urges not necessarily leading to more frequent sex with partners; instead, behaviors like masturbation and promiscuity were common. Emotional formulations ranged from negative to neutral, influenced by whether patients internalized or externalized their hypersexuality. Insight varied, with some patients viewing HS as natural and others seeing it as conflicting with their values. Control over HS was mixed, with efforts to manage behaviors influenced by personal or external factors. The impact on life was predominantly negative, affecting marital closeness, family dynamics, social interactions, work efficiency, and physical health. Patients perceived mostly negative feelings from their partners regarding HS. Stigma was significant, including personal shame, social concealment, and discomfort discussing HS with health professionals. Barriers to seeking help included communication deficits, professional neglect, and stigma. Despite challenges, patients expressed a desire for better guidance and open discussions with health professionals to manage HS. This study uncovered the profound impact of HS on various facets of life such as quality of life, work, and personal relationships, elucidating the emotional distress and societal challenges faced by patients. This preliminary study on hypersexuality in neurological disorders suggests multiple avenues for future research.

**Supplementary Information:**

The online version contains supplementary material available at 10.1038/s41598-024-79966-z.

## Introduction

Impulse-control disorders (ICDs) are a common side effect of dopamine replacement therapy (DRT), especially dopamine agonists (DAs) in Parkinson’s disease (PD)^1,2^. Evidence suggests that ICDs can have a devastating impact on PD patients’ mental health and quality of life^3^.

The most common ICD in PD is pathological gambling^4,5^. Hypersexuality (HS), or compulsive sexual behaviour, has been recognized in recent years as impacting quality of life of patients and their carers^6,7^ and is likely to be underreported. The literature reports varying prevalence figures for HS in PD ranging between 1.9% and 22.8%^8^, partly due to the lack of a validated HS screening questionnaire and the use of the Questionnaire for Impulsive-Compulsive Disorders in Parkinson’s Disease (QUIP) as a mean to quantify HS occurrence^9^.

The behavioural expression associated with hypersexuality involves heightened libido, increased frequency of erection, increased sexually demanding demeanour, seeking sex from prostitutes and use of sex phone lines^10^, changes in sexual orientation^10^, which may be coupled with excessive^10^, compulsive and aggressive masturbation^11–18^, and expression of paraphilias^19,20^, all associated with disrupted frontal inhibition^21,22^. Research suggests two possible reasons for the increased libido: First is the disintegration of the reward circuit, and second is the fact that patients are subjected to perpetual and prolonged dopaminergic system stimulation by medication, which might have functional and structural repercussions^23^.

The exact causes of hypersexuality are poorly understood and demarcated. Reported risk factors include being male, as hypersexuality is exhibited in men more frequently than women^14,24^, younger age at PD onset^23^, and a history of novelty-seeking behaviour^25^. Since smoking and substance abuse have been identified as risk factors for developing impulsive/compulsive behaviours in PD^14,24^, they may be considered as risk factors for hypersexuality also as neither hypersexuality nor any other ICDs were identified by name.

Typically, most hypersexuality cases are associated with dopamine agonists^12,26–28^; however, some studies do report other pharmacological etiologies. A study by Shapiro et al. (2006) describes two PD patients who exhibited hypersexual and paraphilic behaviour following selegiline use^29^. These patients then added dopamine agonists to their medication regimes, which caused them to develop obsessive-compulsive behaviours and punding (a fascination with performing mechanical tasks) behaviours^29^. Another case study by Simonet et al. (2016) describes hypersexuality developing following rasagiline use^30^. Rasagiline and selegiline are both monoamine oxidase-B inhibitors^31^. Further, some research reports hypersexuality developing in patients taking levodopa monotherapy^26,32^, as well as combined levodopa and dopamine agonist therapy^32,33^. Although hypersexuality in PD is most closely associated with pharmacology, it has also been reported in cases following deep brain stimulation of the subthalamic nucleus (STN DBS)^34^ and pallidotomy^35^.

There is very limited research pertaining to hypersexuality-specific behavioural or psychological management options for patients with PD. Recent neuroimaging studies exploring the neural correlates of HS in PD show that sexual visual stimuli trigger a dysfunction in the inhibitory network through abnormal enhanced activation of the mesocorticolimbic pathway (anterior cingulate cortex (ACC), ventral tegmental area (VTA), and caudate) and reduced connectivity between the pre-supplementary motor area (pre-SMA) and the caudate nucleus^36,37^.

The available literature on HS in PD is entirely quantitative in nature, which poses a major limitation to the understanding of the clinical profile of HS in PD and, as a result, leads to inadequate patient-centered care for PD patients. Therefore, a qualitative study stands as an imperative endeavor for multifaceted reasons. Firstly, a qualitative approach provides an in-depth exploration of the phenomenology of HS, unraveling the intricate lived experiences, triggers, and coping mechanisms of individuals affected by this phenomenon. This method enables a nuanced understanding of the qualitative aspects of HS manifestation, shedding light on its diverse expressions. Secondly, the profound impact of HS on the quality of life, work, and personal relationships necessitates a qualitative lens to uncover the emotional distress, disruptions in daily functioning, and social challenges faced by both patients and caregivers. Beyond numerical metrics, this approach captures the holistic impact of HS on various life domains. Finally, by offering a longitudinal perspective, qualitative studies surpass single cross-sectional analyses by illuminating the evolving nature of HS experiences over time. Through prolonged engagement with participants, these studies reveal the trajectory, adaptations, and coping strategies associated with HS in PD patients, thus enriching our comprehension beyond prevalence figures and providing a comprehensive understanding of its clinical, psychological, and sociocultural dimensions.

The objective of this study therefore was to qualitative techniques to systematically gain insight into the clinical phenomenology of HS in PD and its associated clinical, pharmacological, behavioural, cognitive, and psychological factors, as well as its impact on the lives of PD patients.

## Methods

### Study design

This study is a phenomenological qualitative research design study. It employed semi-structured interviews to examine hypersexuality and its impact among nine English-speaking individuals with PD. The study was approved and overseen by UCL Research Ethics Service (Integrated Research Application System [IRAS] ID: 153823) and conducted in accordance with the Declaration of Helsinki. Patients provided written informed consent prior to their participation.

This research is part of a broader UCL project examining hypersexuality in neurological disorders, which includes a recently published systematic review^38^.

### Participants

#### Inclusion/exclusion criteria

Inclusion criteria included being 18 years or older, having clinically diagnosed PD according to the UK Brain Bank Criteria^39^, indicated hypersexuality either in the past or present since developing PD, no cognitive impairment, and an ability to provide informed consent. Exclusion criteria included hypersexuality predating the onset of PD, co-existing neurological disorders as determined by clinical history, secondary causes of Parkinsonism, and difficulty understanding/speaking English.

Hypersexuality was defined as a marked increase in sexual urges, behaviors, or preoccupations that emerged or intensified after the onset of PD. This definition was based on self-reported symptoms and clinical assessment. Additionally, participants needed to have no cognitive impairment and be able to provide informed consent.

#### Recruitment process

**Fig. 1:**
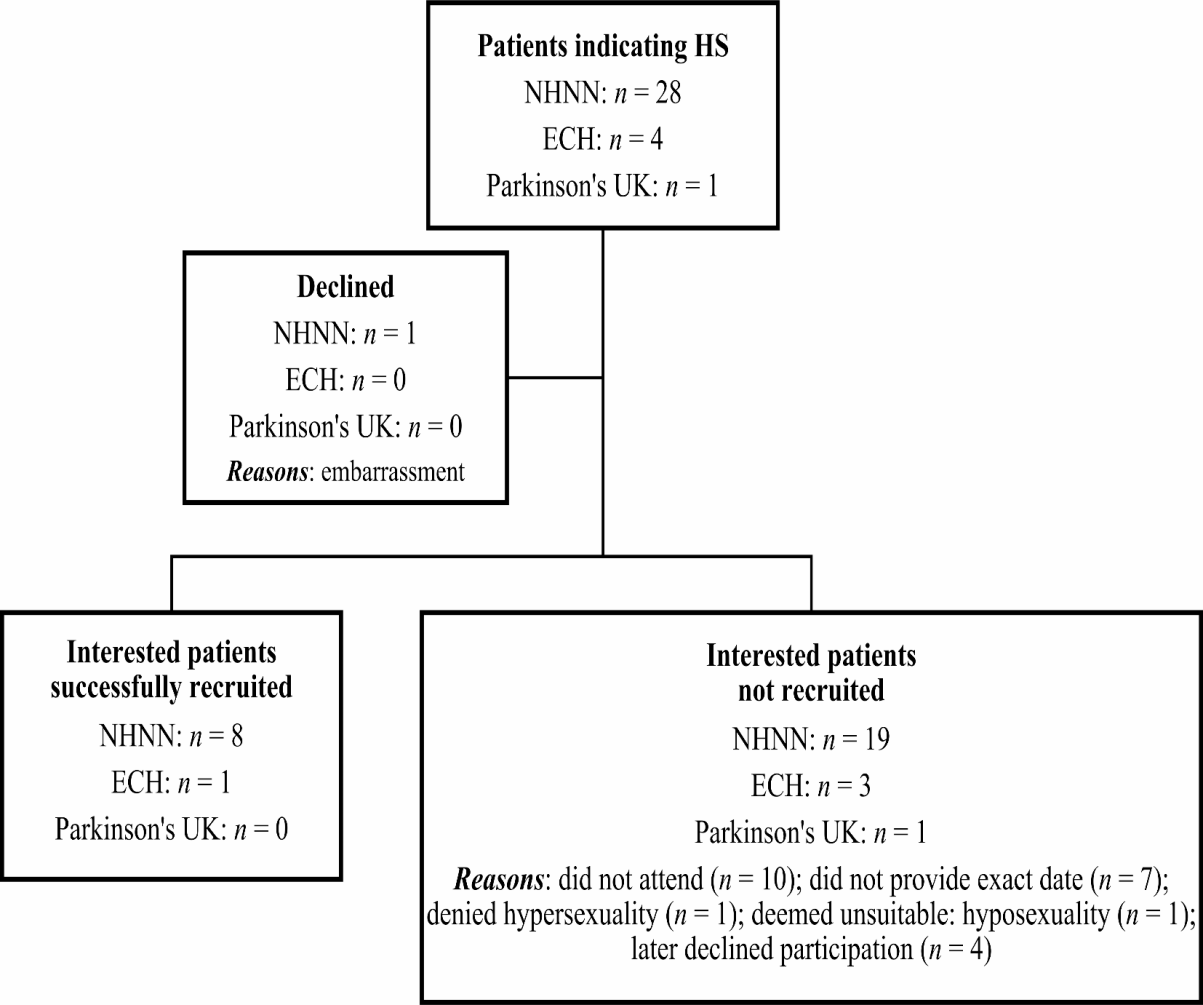
Summary of recruitment results for Parkinson’s disease patients. ECH: Edgware Community Hospital; HS: hypersexuality; NHNN: National Hospital for Neurology and Neurosurgery.

Figure 1 illustrates the recruitment process of our sample. A total of thirty-three patients with PD indicated hypersexuality as having been or still being an issue, twenty-eight of them from the National Hospital for Neurology and Neurosurgery (NHNN), four from Edgware Community Hospital (ECH), and one from Parkinson’s UK.

After indicating hypersexuality during their clinical appointments, twenty-eight patients from the NHNN were initially informed about the study by their consultant neurologists or neuropsychologists. Twenty-seven of them initially agreed to participate and one refused because he believed it would be too embarrassing for him to take part. Of the twenty-seven patients who expressed interest, only eight were successfully recruited for the study. The remaining nineteen either did not attend on the scheduled assessment date, did not provide an exact date to partake, later declined participation after initially agreeing, or were deemed unsuitable due to having hyposexuality rather than hypersexuality. Several attempts were made by the research student and primary supervisor to contact these patients to no avail.

During meetings held with the Parkinson’s Service Lead and the rest of the Parkinson’s Day Unit team at ECH, the necessary protocol was developed and the QUIP was considered, approved, and circulated at the Parkinson’s Day Unit over a period of six months^1^. A leaflet briefly describing the study was attached to each questionnaire. After leaving their contact details on the QUIP, as was requested if they were interested to take part in the study, four patients from ECH were contacted by NT and informed about the study. Of the four patients who expressed interest, only one was successfully recruited for the study. The remaining three patients either later declined participation or later denied having hypersexuality. One of the patients refused because his wife was unhappy for him to take partake. Several attempts were made to contact these patients to no avail.

After being contacted and provided verbal consent, the one patient from Parkinson’s UK did not attend on the scheduled assessment date.

In total, nine PD patients took part in the study.

Interested patients were asked to come to the Department of Uroneurology at the NHNN, where they received written information about the study, signed consent forms, and underwent assessment. Participants received Participation Information Sheets outlining the study’s aims, methods, and potential risks and benefits. Risks included discussing sensitive topics related to sexual behavior, with the option to refuse answers or withdraw without affecting medical care. Any disclosures that posed risk to participants or others, or required by law, would be reported to authorities. Benefits were framed as contributing to a better understanding of hypersexuality in neurological disorders, potentially leading to improved care and psychological interventions. Following consent, patients were assessed using a semi-structured interview designed to evaluate their experiences with hypersexuality and its impact.

NT conducted the main one-to-one interviews. However, help was on hand (JNP) in case interviewer required assistance during the course of the interview.

### Sample size

The sample size for this qualitative study was nine patients, which is considered adequate for exploratory research in qualitative methodologies. In qualitative research, the focus is on in-depth understanding rather than statistical generalizability. The concept of “saturation” was used as a guide, defined as the point where additional data no longer contribute new insights to the research questions^40,41^. According to qualitative research standards, a sample size of 9 can be sufficient for generating meaningful insights, especially in studies involving sensitive topics like hypersexuality in PD. This number allows for a thorough exploration of individual experiences and contributes to theory development within the constraints of qualitative research.

### Interview methodology

The nine patients took part in face-to-face qualitative interviews conducted by a trained interviewer. The interviews ranged from two hours to nearly four hours in duration. Written informed consent was obtained from all patients prior to the interview. The interviewer used the Patient Assessment Interview (Appendix A), a semi-structured thirty-item interview that was used to assess patients’ experiences with hypersexuality and its impact. Due to the sensitive nature of the issue being explored, the interviewer was responsible for modifying the interview questions as was deemed fit to alleviate any possible discomfort or uneasiness of participants.

### Qualitative analysis

All interviews were audio-recorded and transcribed verbatim and were analyzed (NT) using thematic analysis methods^42^. Transcription was unavailable for 2 patients because they declined having the Dictaphone record their interviews. The data collected, therefore, was based on thorough note-taking during the interviews for each question, which included some verbatim quotations. After all the participants’ data were written up, several readings of the data were carried out by the interviewer and two independent researchers. The participants’ responses to each interview question were entered into an Excel spreadsheet = and the researchers began identifying extracts, either through annotation or highlighting, from the participants’ responses that could be combined to reflect specific ideas, words, and patterns. After the data was collated and coded, the researchers compared and discussed codes, established coherent connections between them, and consequently categorized them within appropriate themes. After initial themes were generated, they were reviewed and kept, modified, or removed. Findings were summarized and conclusions drawn.

## Results

### Demographic and clinical characteristics

A total of nine patients with PD (six males; mean age 61.7 ± 13.3 years between 44 and 78 years – three females; mean age 64.3 ± 5.7 years between 58 and 69 years) participated in this study. The mean age of onset of PD was 51.4 ± 12.5 and ranged between 31 and 68 years. The mean age of onset of hypersexuality was 54.1 ± 11.5 and ranged between 35 and 68 years. Eight of the nine patients were in monogamous relationships, and one was single. In regards to sexual orientation, eight patients identified as being heterosexual, and one patient as homosexual which was a change in (Table 1).

**Table 1:** Patient sample descriptives (*N* = 9).

Variable	Patient 1	Patient 2	Patient 3	Patient 4	Patient 5	Patient 6	Patient 7	Patient 8	Patient 9
Gender	Male	Male	Male	Female	Female	Male	Female	Male	Male
Age (year)									
Onset of PD	31	37	68	52	58	42	54	56	65
Onset of hypersexuality	35	42	68	55	63	44	54	61	65
At assessment	44	47	78	58	69	66	66	68	67
In a relationship	No	Yes	Yes	Yes	Yes	Yes	Yes	Yes	Yes
Sexual orientation	Homosexual	Heterosexual	Heterosexual	Heterosexual	Heterosexual	Heterosexual	Heterosexual	Heterosexual	Heterosexual
Past sexual abuse	Molested by uncle; aged 3	No	No	No	Inappropriately touched by tennis coach; aged 8–9	Inappropriately touched by stranger in local forest; aged 8	No	No	No
Past addictions	None	None	None	None	Alcohol	None	None	None	None
Past cognitive or behavioural disorders	None	None	None	None	None	None	None	None	None
Medications when hypersexuality first indicated in clinic*	Ropinirole Amantadine Fesoterodine Pregabalin	Ropinirole Amantadine Selegiline Madopar Stalevo	NA**	Stalevo Rotigotine patch Azilect Clonazepam	Ropinirole Rasagiline Entacapone Amantadine	Ropinirole Madopar Rasagiline Amantadine	Ropinirole Madopar Citalopram	Ropinirole Madopar Stalevo	Ropinirole Sinemet Escitalopram
Medications at the time of assessment	Stalevo Trihexyphenidyl Amantadine Clonazepam Detrusitol Fluoxetine	Stalevo Madopar Amantadine Selegiline Movicol	NA**	Stalevo Rotigotine patch Rasagiline	Ropinirole Stalevo Amantadine	Ropinirole Madopar Rasagiline Amantadine	Madopar Duloxetine	Ropinirole Madopar Stalevo	Ropinirole Sinemet
Patient perception of implicating medication	Ropinirole	Ropinirole	Unsure (Sinamet or Selegiline)	Rotigotine	Rasagiline	Ropinirole	Unsure (Ropinirole)	Ropinirole	Ropinirole
Implicating medication reduced/ discontinued*	Yes discontinued	Yes discontinued	No	Yes reduced	Yes discontinued	No	Yes discontinued	No	Yes reduced
Hypersexual at assessment	Yes	Yes	Yes	Yes	Yes	Yes	Yes	Yes	Yes
Currently hypersexual*	Yes (not as severe)	Yes	NA**	Yes	No	Yes	Yes	Yes	Yes
DBS*	No	Yes	No	No	No	Yes	No	No	No
Type	–	Bilateral STN	–	–	–	Bilateral STN	–	–	–
Consented to Dictaphone	Yes	No	Yes	Yes	Yes	Yes	No	Yes	Yes
Partner took part in study	No partner	Yes (Carer 2)	No	No	Yes (Carer 4)	No	Yes (Carer 3)	Yes (Carer 5)	No
Completed full set of assessments	Yes	Yes	Yes	Yes	Yes	Yes	No	Yes	No
Associated symptoms									
Sexual behaviour	Preoccupation with sex Increased desire for sex Change in sexual orientation Pornography Having sex more frequently Prostitutes Promiscuity Bath houses Saunas Prostitutes	Increased desire for sex with wife Having sex more frequently Increased masturbation Pornography	Preoccupation with sex Increased desire for sex with girlfriend Having sex more frequently	Preoccupation with sex Increased desire for sex Having sex more frequently Increased masturbation Extramarital affair	Preoccupation with sex Increased desire for sex with husband Having sex more frequently Lust for therapist Increased masturbation	Preoccupation with sex Increased desire for the female body Increased masturbation Increasingly flirtatious with other women Pornography Massage parlors Extramarital affair	Pornography Insatiable desire for masturbation	Increased masturbation Increased desire for sex with wife	Increased desire for sex Preoccupation with sex Pornography Exhibitionism Massage parlors Naturist clubs
Other impulse control disorders	Compulsive gambling Compulsive eating Compulsive buying	None	None	Compulsive buying	Compulsive eating	Compulsive eating Compulsive buying	Compulsive eating Compulsive buying	None	Compulsive eating
Other compulsive behaviours	Constructing Exercising	Listening to music Constantly rearranging lounge Redoing jobs that do not need doing	Cleaning the house	Running Jumping Decluttering and tidying the house Planning unrealistic things	Trying to put things right around the house Working on anything that needed fixing	Playing music Painting	None	None	None

DBS: deep brain stimulation; STN: subthalamic nucleus

*. Information obtained from patient’s clinical notes.

**. Information could not be obtained due to inability to access clinical notes at ECH.

### Qualitative thematic analysis

Ten themes emerged as a result of the analysis: Clinical manifestations, sexual practices, emotional formulations and attributions, insight, control, impact, perceptions about partners’ feelings, stigma, professional help-seeking barriers, and aspirations.

## Theme 1: Manifestations

This theme delineates the diverse expressions of hypersexuality across patients and highlights some of the commonalities between them.

### Cognitions

Regarding patients’ cognitions, a marked increase in sexual preoccupation emerged, along with heightened desires for sexual activity and engagement with others, to the extent that their preoccupation affected their daily living.

*“It’s a recurrent thing that you think about … on the way to work this morning I was thinking ‘I could go to my naturist place tomorrow at three o’clock and should I tell my wife where I’m going?’…”* (Patient 9).

*“I was never late but the thing is the quality of my work…”* (Patient 1).

*“It affects my social life because I’m always thinking about it… I choose to stay at home…”* (Patient 3).

### Behaviours

Furthermore, hypersexuality induced alterations in pre-existing behaviours or the development of different, unusual, risk-taking behaviours and a preoccupation with sex. This was evident through lack of adherence to other commitments, obsessive thoughts, and strategic planning for sexual encounters. In one of the most extreme forms of new risky behaviours, Patient 1, who had identified as a homosexual his entire life and had formerly been in an eight-year monogamous relationship with a man, recalled being in a “hyper” state with a newly-developed unusual sexual interest in women and call girls, as well as an unusual increased desire for sex with men. He recounted spending six hours a day in gay bath houses and saunas, sleeping with up to fourteen to fifteen strange men a day. This patient also admitted having been careless and indiscriminate about his sexual pursuits and having had multiple sexual partners at once without asking about the status of HIV and other sexually transmitted diseases. Like Patient 6 who also reported visiting massage parlours, Patient 1 admitted to paying a lot of money for sex, something he did not do before the onset of his hypersexuality.

Patient 9 also spent considerable time on “happy endings” in massage parlours, as well as developing other new unusual behaviours. He was the only patient who developed paraphilic behaviours after developing hypersexuality:

*“I’m interested in things that I was never interested in before… exhibitionism… one day I took all my clothes off and lay down in the garden… and since then… I have been to a few nude beaches… and then… I started thinking ‘I wonder what these massage parlours are like’… so I had to fight with myself… I went in… you get a proper massage if you choose somebody carefully… and then they have this formula… they say ‘Would you like any extras?’ and one day I said yes… I also go sometimes to this naturist club…”* (Patient 9).

### Preoccupation

Preoccupation is characterized by excessive thinking about something to the extent that it impacts daily living. Most patients reported that their preoccupation with sex significantly disrupted their daily lives, with six out of nine describing how it affected their adherence to commitments, obsessive thoughts, and planning for sexual activities, leading to neglect of relationships, work, and other daily tasks.

*“I was never late but the thing is the quality of my work…”* (Patient 1).

*“It affects my social life because I’m always thinking about it… I choose to stay at home…”* (Patient 3).

### Compulsivity

This preoccupation with sex often manifested as compulsive sexual behaviour, although not all patients indicated this (though it was suggested). One homosexual participant (Patient 1) explained that his visits to bath houses and saunas could take up to six hours of his day and often involved having sex with multiple men at once:

*“I sleep five o’clock in the afternoon… that’s when I come back from… sex… I sleep five in the evening… I wake up twelve in the midnight and put on my jacket and go back…”* (Patient 1).

Lastly, negative emotions such as sadness, depression, loneliness, anger, frustration and “OFF” states in PD appeared to trigger hypersexual episodes in patients.

*“I’m not sure what it is… it’s probably frustration… anger…”* (Patient 6).

*“I would say when I’m depressed… when I’m sad… when I have no one to talk to…”* (Patient 1).

## Theme 2: Sexual practices

This theme discusses the impact of hypersexuality on patients’ sexual behaviours. The participants’ heightened sexual urges led to various types of sexual practices, which can be categorized as practices with their partners, with themselves, with others, and deviant sexual practices.

### Practices with the partner

It was observed that participants’ increased desire for sex due to hypersexuality did not consistently result in an increased frequency of sexual activity with their partners. In fact, most patients reported either a reduced or similar frequency of sexual engagement with their partners compared to their pre-hypersexuality frequency, with only a minority noting an increase. Of the patients who reported increased frequency of sex with their partner, one (Patient 2) explained that this had eventually tapered off due to tension in his relationship with his wife, while in contrast the other indicated that a high level of sexual activity had continued with her willing participant husband, including watching pornography together (Patient 5).

### Practices with themselves

Furthermore, patients frequently engaged in sexual practices with themselves, such as masturbation and use of pornographic materials, although the extent of masturbation as a sexual activity varied among patients. Two patients who reported that they rarely or never masturbated were those who were indulging in quite promiscuous and risky sexual behaviours and therefore expressed the view that they had no need to pleasure themselves.

*“I’ve stopped that… I find it a bit boring now… but as I say it’s all of ordinary people doing ordinary things… so after a while you think… it’s not very exciting…”* (Patient 9).

In contrast, Patient 7’s hypersexuality seemed to have manifested solely in an increase in masturbation, which she was indulging in up to ten times a day. She claimed that “*the more [she] did it*,* the more [she] wanted it… [and] had to stop to get work done…*”. Although this patient claimed that the “*sense of release excites [her]*”, she also reported that she did not feel satisfied from masturbating because it made her want it more. In the case of five patients (1, 2, 6, 7, and 9), the use of pornographic materials appeared to develop or intensify following the onset of hypersexuality.

### Practices with others

Patients also engaged in sexual practices with others, encompassing promiscuity, anonymous sexual encounters, paying for sexual services, and involvement in extramarital affairs.

*“You have to understand… bath houses and sauna… in certain days you get like place full with at least two hundred and… us Arabs quite wanted… everybody all over you… I started to have sex with fourteen… fifteen person a day…”* (Patient 1).

*“There’s something terribly alluring about you walk into this little room with somebody you’ve never seen before and you take off all your clothes and you lie down flat and she rubs your body… I mean for God’s sake… that’s a pretty exciting sexual thing…”* (Patient 9).

### Deviant practices

Deviant sexual practices included paraphilic behaviours. Hypersexuality did not necessarily translate into paraphilic behaviour as one patient indicated exhibitionism, which can be defined as the act of showing one’s genitals in public:

*“I suddenly… it wasn’t really a change in sexuality… it was in exhibitionism… so one day I took all my clothes off and lay down in the garden… my wife came out and said ‘What are you doing? The neighbours can see’…”* (Patient 9).

## Theme 3: Emotional formulations and attributions

This theme focuses on the emotional formulations and attributions that patients formed regarding their hypersexuality.

### Internalization of hypersexuality and negative feelings

Evidently, if the hypersexuality was internalised (blaming development of the behaviour on themselves), patients tended to develop negative feelings about it.

*“The doctor’s reaction … got me really depressed because I didn’t get an answer for any of my worries… about my body …. I felt like I … haven’t been believed… and I started to reflect on my personality. I used to say… I’m just [a] horrible person because I’m doing things no… nobody is doing and I always used to push it back on myself…”* (Patient 1).

### Externalization of hypersexuality and neutral feelings

If the hypersexuality was externalised (regarding behaviour as stemming from external determinants), patients appeared to develop neutral feelings about it. Patient 9 seemingly attributed his hypersexuality to the “chemistry” of Ropinirole and his body’s need for it:

*“I tried coming off the Ropinirole about three to four weeks ago… and it was unbearable… the Parkinson’s just suddenly kicked in and I wasn’t used to it… but I want it back to where it was… I’d rather be hypersexual than slightly weird… it’s better than the alternative … if somebody can put me back on Ropinirole I’d take whatever comes… I’m addicted to Ropinirole it seems…”* (Patient 9).

### No internalization or externalization of hypersexuality and positive feelings

If the hypersexuality was neither internalised nor externalised, patients appeared to develop positive feelings towards it. In the case of this sample, three patients (3, 4 and 5) fell into this category, and reported feelings of happiness associated with their hypersexuality. For example, Patient 3 reported feeling “*happy*”, “*satisfied*” and “*comfortable with it*”, while Patient 4 described feeling a “*kind of high*” as a result of her condition.

## Theme 4: Insight

This theme highlights the findings regarding patients’ level of insight into their hypersexuality. Within the context of hypersexuality, patients demonstrating insight typically exhibit the following attributes: acknowledgment of the presence of the condition, recognition of its unnatural character, partial awareness of its underlying determinants, and a sincere aspiration to mitigate it.

### Acknowledging the presence of the problem

When queried about whether they had observed an escalation in sexual thoughts or behaviors following the onset of PD, all patients indicated awareness of the alteration in their sexual disposition. However, this awareness did not uniformly translate into a perception of hypersexuality as problematic.

### Recognizing hypersexuality as natural or unnatural

Moreover, a majority of patients (*n* = 6) asserted that their hypersexuality was congruent with their personal values, thus perceiving it as natural and in accordance with their individual beliefs. Patients 5 and 9 also expressed views which demonstrated that they were generally comfortable with their hypersexuality and even regretted not being more sexually active before the onset of their condition:

*“I think it’s fine in a way having that much… I mean I… it makes me in a way… discontented… like I should have had more sex in my life…”* (Patient 5).

*“One day I took all my clothes off and lay down in the garden… my wife came out and said ‘What are you doing? The neighbours can see’… and since then my wife and I have been to a few nude beaches… this is all harmless stuff but it’s… especially… you know my wife and I up to this point have been boringly straight… normal… straight… and not interested in anything unusual…”* (Patient 9).

Conversely, a subset of patients held the view that their hypersexuality was unnatural, primarily because it seemed to conflict with their personal values and convictions.

### Beliefs about causes of the problem

Irrespective of their stance on its naturalness, all patients attributed the condition predominantly to the use of dopaminergic medication in the management of PD. The main form of medication believed to have caused hypersexuality was Ropinirole, cited as such by more than half the sample (Patients 1, 2, 6, 8, and 9).

*“I take it like every hour… every two hours because I can see it numbing my… [PD symptoms] … but while it’s numbing my… it’s getting me so hyper…”* (Patient 1).

*“I was on Ropinirole … gave me a buzz… good stuff… used to take it and it would give me an erection…”* (Patient 6).

Patient 2 believed the DBS further exacerbated his perceived Ropinirole-induced hypersexuality, while Patient 6 believed his DBS helped in reducing it. Two of the patients (4 and 5) attributed their hypersexuality to other forms of medication used to manage their PD, such as Rasagiline or Rotigotine patches. The remaining two patients (3 and 7) were less certain as to which medication contributed to their hypersexuality, but expressed the suspicion that this might be the Sinemet or Selegiline (Patient 3), or Madopar (Patient 5).

### Desire to overcome the problem

Finally, the findings classified patients into three principal categories concerning their desire to overcome hypersexuality: a true desire to overcome the problem, an unconvinced/convincing desire to overcome the problem, or no obvious desire to overcome hypersexuality.

## Theme 5: Control

This theme underscores that, despite the patients having limited control over the development of hypersexuality following the administration of dopaminergic medication for PD, some individuals indicated a degree of control over their behaviors, while others expressed a loss of control.

### Loss of control

Only three of the patients (1, 6, and 7) expressed the feeling that they had no control over their hypersexuality, for example as explained by Patient 1:

*“I always think ‘Is it like a habit that I’m living?’… it’s in my life… I keep going back for it… like eating… Because I have it since I was three… it’s something part of my personality… I can’t… I tried … it would be good to know if I can control it more… what’s the methods… is there any particular way out… I’ll do anything just to have that…”* (Patient 1).

In contrast, the rest of the patients all indicated that they felt they could control their condition (The information for this subtheme is limited as it was based upon one “Yes/No” question in the interview.).

### Attempt to reduce/stop

Most patients (*n* = 6) indicated that they had made attempts to reduce/stop their hypersexuality. These patients were categorised into two groups: patients who truly attempted to reduce/stop their hypersexuality due to what might be conceptualised as “internal” factors and patients who attempted to reduce it due to “external” factors. For example, internal factors included the patients’ own feelings about their condition:

*“All the time I just wondered… wondering and asking myself why am I doing it”* (Patient 1).

However, external factors included the views or feelings of other people, such as their partner’s unhappiness with their hypersexuality. For example, Patient 2 reported that he had attempted to abstain from sex only for the sake of his wife’s happiness; he expressed the view that his condition made him sad because sex should really be “*part of a healthy relationship between husband and wife*”.

## Theme 6: Impact

This theme outlines the positive and negative impacts that hypersexuality had on patients’ marital lives, family lives, social lives, work and daily activities, finances, health, mood, sleep, self-confidence, and quality of life. Findings indicated that hypersexuality negatively impacted at least one of the different areas of living for each of the patients.

### Marital life

The majority of patients reported negative consequences in their marital lives, particularly with respect to the emotional closeness and intimacy within their marriages. This negative impact seemed to stem from two distinct factors. In some cases, the increased demand for impersonal or mechanical sexual encounters had caused tension, leading to breakdowns in communication and sexual relations, infidelity, or a lack of focus on their partners. Additionally, some patients were aware of how their behaviors made their spouses feel, resulting in negative emotions such as anger, embarrassment, disapproval, betrayal, and sadness.

*“I might touch her… and that might annoy her because I’ve done it in the kitchen while she’s doing something… I want to touch her… but… she doesn’t like me touching her in the kitchen… for her the bedroom is the place…”* (Patient 3).

Interestingly, only one patient indicated that hypersexuality had an indirectly positive impact on her marital life by acting as a catalyst for her and her husband to seek counselling.

*“I think what it’s done is that it’s highlighted the lack of communication between [us]… so in a way not negative… in a way it’s positive… in fact to the extent that I’ve arranged to have counselling to sort it out…”* (Patient 4).

### Family, social life and daily activities

Negative impact on family life included neglect of family members, more absence, and more irritability. Negative impact on social life included neglect and avoidance of socializing due to preoccupation with sex.

*“I started to ignore best friends and I start to ignore people in general…”* (Patient 1).

Negative impact on work and daily activities included decrease in efficiency and a lack of concentration on work due to preoccupation with sex. Negative impact on finances included spending large sums of money on call girls and in massage parlours. Negative impacts on physical health included sexually transmitted diseases, as well as loss of appetite due to preoccupation with sex. Patient 1 was the only participant who indicated that hypersexuality had negatively affected his health. He explained: “*I caught lots of… stuff like flu… bad flu… and I had horrible sore throat…*”. He had also caught sexually transmitted diseases and had an HIV scare. “*I think I had the… not herpes the other one… chlamydia or gonorrhoea…*”. Further, he claimed he felt anorexic as a result of only drinking milk and hot chocolate due to his preoccupation with sex.

### Health and well-being

Negative impact on mood included feelings of depression, stress, unhappiness, and anxiety. For example, Patient 1 explained that his hypersexuality caused him to feel depressed and very stressed. Patient 2, who reported being stressed, worried, and “*on the verge of depression*” indicated that this was mainly due to his wife’s unhappiness with his hypersexuality, which had a negative impact on his own feelings, while Patient 8 similarly explained that his wife’s rejection of his sexual advances made him “*moody*” and unhappy. The interviews also suggested that as a result of their hypersexuality – or perhaps the medications which were also causing this – the emotions of many of the patients were heightened or unstable and fluctuating. As Patient 4 explained “*Everything is more heightened so if I’m upset then I’m really upset…*”, while Patients 5, 6, and 7 also reported fluctuating emotions.

Negative impact on sleep included staying awake due to preoccupation with sex and planning next sexual endeavours. Self-confidence, on the other hand, was equally positively and negatively impacted with some patients feeling more sexually attractive and others reported lower self-confidence due to attitudes of their wives towards their hypersexual behaviour.

Overall, the prevailing sentiment among most patients was that hypersexuality had a predominantly negative effect on their lives.

## Theme 7: Perceptions about partners’ feelings regarding hypersexuality

This theme delves into the patients’ perceptions of their partners’ feelings regarding hypersexuality. Approximately half of the patients conveyed that their partners held negative sentiments toward their hypersexuality. In general, these patients appeared to be sad or upset by their partners’ attitudes towards their condition.

*“It makes me feel rejected… I find it more difficult as time goes by to go back into the situation and having a conversation about the problem… I’m a coward like that I suppose…”* (Patient 8).

*“I initiate conversation about it… she puts it in a little box and locks the key and never thinks about it I’m sure… and when I raise it she says ‘Oh for God’s sake do we have to talk about it?’”* (Patient 9).

In general, the patients appeared to experience sadness or distress in response to their partners’ attitudes toward their condition. Conversely, a minority of patients reported a more lighthearted or understanding response from their partners regarding the condition. This may suggest a greater degree of comprehension of their behaviours, even if these behaviours were occasionally viewed as unwelcome or excessive. However, it is important to note that these patients constituted a minority, and the prevailing sentiment among most patients was that their hypersexuality had a negative impact on their partners.

*“I mean essentially he is amused by it you know… he wanted to see what it was… it is artificial in a way… I think he is a bit bemused…”* (Patient 5).

*“She ribs me… she jokes about it…”* (Patient 3).

## Theme 8: Stigma

This theme illuminates prevalent stereotypes held by patients regarding hypersexuality, delineating three distinct dimensions of associated stigma: personal, social, and help-seeking.

### Personal stigma

Personal stigma manifests in sentiments of shame and guilt, possibly arising from the pervasive sensitivity and societal taboos surrounding matters of sexuality. For example, Patient 1 appeared to be guilt-stricken as he described feeling like a “*horrible person because I’m doing things… nobody is doing*”. Patient 7, on the other hand, felt “*ashamed*” because she was raised in a Victorian household with Jewish upbringing that believed that the act of masturbation, which she enjoyed doing, was “*dirty*”.

Additionally, it surfaces in references to gender, wherein male patients may seek to normalize their hypersexual inclinations. Furthermore, older individuals may find it particularly uncomfortable to broach discussions regarding sexuality in the context of advancing age.

*“You know I’m sixty-seven for God’s sakes… isn’t it time I knew… I should’ve known and done everything there is to do by the time I’m sixty-seven…”* (Patient 9).

*“It’s not easy for people to discuss… I don’t find it easy… not really… I think if you are in the older range it would be worse…”* (Patient 8).

### Social stigma

Social stigma is observable as patients endeavor to conceal their experiences with hypersexuality, apprehensive of potential revelation to others.

*“I always do… I don’t talk about it… to anyone…”* (Patient 1).

*“Nobody knows about it… except my oldest friend… he knows something’s wrong and I’m going to talk to him on Friday…”* (Patient 9).

*“If I walked out of this naturist club straight into the arms of a friend I’d be so embarrassed… it would be terrible… I just worry that people will think I’m a weirdo…”* (Patient 9).

This phenomenon was notably palpable in instances during interviews wherein patients exhibited uneasy and inappropriate laughter when confronted with sexually-specific inquiries or when elaborating on their intimate encounters and practices.

### Help-seeking stigma

Help-seeking stigma is discernible in select male participants expressing a marked preference for conferring about their hypersexuality with male physicians, evincing discomfort or embarrassment in broaching the subject with female practitioners. In explaining this, Patient 2 demonstrated a stereotypical perception of women who “*all think the same way*” and expressed the view that a male doctor would understand him better. Patient 6 reported that he had not told his GP about his hypersexuality because “*she’s a female doctor*”. This inclination underscores a broader hesitancy within the patient-physician dynamic. These findings underscore the persistent boundaries surrounding discussions of sexuality, rendering the discourse on hypersexuality inherently challenging.

## Theme 9: Professional help-seeking barriers

This theme highlights the challenges encountered by patients in their pursuit of information concerning hypersexuality from healthcare practitioners. The findings reveal a multifaceted array of obstacles, encompassing deficient communication, inadequate comprehension, limited educational resources, instances of professional neglect, prevailing stigma surrounding hypersexuality, and the inherent difficulties associated with broaching discussions pertaining to sexuality.

Patient 2 explained that the PD nurses at the hospital “*did not know much about it*”, although he had brought it up to them, and “*didn’t dig deeper*”, while the lack of helpful information from his GP exacerbated Patient 1’s negative feelings about himself.

*“The doctor’s reaction … got me really depressed because I didn’t get an answer for any of my worries… my inquiry… for my worries about my body and I felt like I… either neglected or denied or… haven’t been believed… I thought… and I started to reflect on my personality… I used to say I’m an evil… I’m anonymous… I’m just horrible person… I’m just horrible person because I’m doing things no… nobody is doing and I always used to push it back on myself…”* (Patient 1).

*“Did the psychiatrist know anything more than I knew? I don’t think so… hypersexuality has not crossed the threshold where people can talk about it… it’s really embarrassing to discuss… feels horrible being at consultation where everything is being discussed but this… the doctors don’t know what to do…”* (Patient 7).

These combined factors collectively hindered patients’ capacity to solicit guidance, thereby acting as deterrents to their proactive engagement with healthcare providers on this matter.

## Theme 10: Aspirations

This theme highlights some of the patients’ expressed aspirations to help them move forward with hypersexuality, although this was not directly asked.

### Professional help-seeking

Patients’ pronounced urgency for information and counsel from healthcare practitioners is strikingly evident. Broadly, participants underscore a paramount need for healthcare professionals to warn their patients about hypersexuality, elucidate its phenomenology and manifestations, and extend assistance to those grappling with its impact. A prevailing sentiment among the majority of patients pertains to a desire for a better understanding of their hypersexuality, and be able to discuss it openly with health professionals without fear or embarrassment. Patient 2 expressed needing “people to be more decisive in their opinions” rather than just having a platform to “*talk*”.

### Marital help

Regarding marital support, select patients expressed aspirations to improve their relationships. These inclinations materialized through a proactive pursuit of counseling sessions and an articulated wish to adapt their sexual conduct to align with their partners’ comfort levels. For example, Patient 4 hoped that counselling would help her overcome the issues in her marriage. She claimed that although she did “*not necessarily*” want to overcome her hypersexuality, she did want to “feel comfortable talking about it with [her] husband”.

### Gaining control

Moreover, a subset of patients articulated, through various means, a keen desire to exert enhanced agency over their hypersexuality. This was expressed most directly by Patient 1, who claimed he had no control over his hypersexuality:

*“I need to know… it would be good to know if I can control it more… what’s the methods… is there any particular way out… I’ll do anything just to have that…”* (Patient 1).

Table 2 outlines the labels of each theme and a summary for each.

**Table 2:** Labels and summaries of themes for patients.

Label of theme	Summary
***Manifestations***	The study detailed various manifestations of hypersexuality among patients, including increased preoccupation with sex, heightened desire for sexual acts and external partners, and altered behaviours, such as risk-taking and obsessive thoughts. These changes disrupted commitments, reflecting obsessive planning for sexual encounters and occasional engagement in compulsive sexual behaviour. Additionally, the findings suggested a connection between negative emotions and the triggering of hypersexual episodes in patients.
***Sexual practices***	The study delineated how hypersexuality impacted patients’ sexual behaviours across different domains. While increased sexual urges were evident, this did not consistently result in heightened frequency of sex with partners; instead, most patients reported either decreased or unchanged frequency. Patients engaged in various practices, including masturbation and increased use of pornographic materials, with a wide range in the reported extent of masturbation as a sexual activity. Furthermore, the study revealed diverse behaviours with others, such as promiscuity, anonymous encounters, payment for sex, and extramarital affairs, indicating a varied response to hypersexuality among individuals. Additionally, the findings indicated that hypersexuality did not universally lead to paraphilic behaviour among the patients studied.
***Emotional formulations and attributions***	The study revealed distinct emotional responses and attributions regarding hypersexuality among patients. Those internalizing hypersexuality, blaming themselves, tended to harbour negative feelings. Externalizing the behaviour, attributing it to external factors, led to neutral emotional responses. Patients neither internalizing nor externalizing hypersexuality tended to develop positive feelings toward it, indicating varying emotional formulations based on internal or external attributions of the condition.
***Insight***	The study revealed diverse patient perceptions regarding their hypersexuality in Parkinson’s disease. While all acknowledged increased sexual behaviour post-Parkinson’s onset, not all considered it problematic; over half viewed it as natural aligning with personal values, while the rest saw it as conflicting with their beliefs. Despite varied perceptions, patients attributed hypersexuality primarily to dopaminergic medication, categorized into three groups based on their commitment to address the issue: those genuinely motivated, uncertain, or displaying no clear desire to overcome hypersexuality.
***Control***	The study highlighted patients’ varied control over hypersexuality onset post-dopaminergic medication for Parkinson’s disease management. While some asserted control over their behaviour, others expressed a sense of losing control. Most patients attempted to reduce or halt hypersexuality, categorized into two groups: those motivated by internal factors (their personal feelings about the condition) and those influenced by external factors (such as partners’ discontent with their hypersexuality).
***Impact***	The study delineated multifaceted negative impacts of hypersexuality on various facets of patients’ lives. Hypersexuality detrimentally affected marital closeness, causing tension due to impersonal or mechanical sexual demands or infidelity, triggering emotions like anger, embarrassment, and sadness in spouses. It negatively impacted family dynamics through neglect and increased irritability, hindered social interactions, decreased work efficiency and concentration, and led to financial strain from excessive spending on sexual services. Additionally, it caused physical health concerns like sexually transmitted diseases, heightened emotional instability, disturbed sleep due to preoccupation with sex, and fluctuating self-confidence, contributing collectively to a predominantly negative impact on patients’ overall quality of life.
***Perceptions about partners’ feelings regarding hypersexuality***	The study unveiled patients’ perceptions of their partners’ attitudes toward hypersexuality, with approximately half noting negative feelings from their partners. Many reported their spouses’ reluctance to discuss the condition, leading to discomfort in addressing it themselves, evoking feelings of sadness or upset among the patients. Contrarily, a few patients indicated a lighter, more understanding response from their partners, albeit in the minority, as most acknowledged a predominantly negative impact of hypersexuality on their partners.
***Stigma***	The study identified three stigma forms associated with hypersexuality: personal stigma linked to shame and guilt related to societal taboos around sex, social stigma evidenced by patients’ concealment efforts and discomfort during sexually explicit discussions, and help-seeking stigma seen in preferences for discussing hypersexuality with male doctors and discomfort with female doctors, revealing societal barriers hindering open discussions on hypersexuality. These findings underscore the challenges posed by societal norms and taboos that complicate discussions about hypersexuality.
***Professional help-seeking barriers***	The study revealed multifaceted challenges faced by patients seeking information about hypersexuality from health professionals, including deficient communication, limited understanding, inadequate education, professional neglect, stigma surrounding hypersexuality, and the discomfort in discussing sexual matters, collectively obstructing patients’ access to advice and deterring their attempts to seek help.
***Aspirations***	Patients expressed a strong desire for guidance and information from health professionals regarding hypersexuality, yearning for explanations about its nature, manifestations, and practical assistance for coping with it. They emphasized the necessity for open discussions without embarrassment or fear, indicating a willingness to comprehend hypersexuality better. Some patients expressed aspirations to enhance their relationships by seeking counselling sessions and modifying their sexual behaviour to align better with their partner’s comfort. Additionally, many patients sought ways to gain better control over their hypersexuality, reflecting a proactive stance toward managing their condition.

PD: Parkinson’s disease

## Discussion

In PD research, hypersexuality is often discussed in conjunction with other impulse control disorders, lacking comprehensive examination in isolation. Existing studies focus more on sexual dysfunction such as erectile dysfunction and hyposexuality^43^ rather than hypersexuality. While recent years have seen increased recognition of hypersexuality as a research concern, information on its manifestations, impact, and correlations remains scarce. This scarcity hampers the ability to compare results with similar data for different patient populations. The aim of this study was to utilise qualitative research methodology to systematically evaluate the clinical phenomenology and impact of hypersexuality on patients with PD. Although the research is focused on sexual behaviour and sexuality, which are sensitive topics, it was important to systematically explore hypersexuality to (1) inform the understanding of the factors and characteristics of hypersexuality and the ways it can manifest (2), explore the impact hypersexuality has on patients to help raise awareness about the issue, and consequently (3) help mitigate the stigma surrounding sex.

The findings of our study suggest that hypersexuality manifests across PD patients in terms of thoughts and behaviours. Sexual changes were observed in patients’ sexual cognitions and behaviours. The changes include but are not limited to the preoccupation with sex, change in sexual orientation, commenting about other women’s underwear, pretending to be wife on dating sites, and transvestic fetishism. These changes can be summarised into five categories: (1) Increased sexual urges/thoughts/fantasies/frequency of sexual acts (2), Self-stimulating sexual behaviour/interests (3), Compulsive/impulsive sexual behaviour (4), New sexual interests/behaviours (e.g. paraphilias and change in orientation), and (5) Illegal sexual behaviour. Similar themes were reported in a systematic review by Codling et al. (2015) documenting deficits in learning from negative outcomes, yet ultimately acknowledging insufficient data to draw definitive conclusions^47^. Furthermore, the literature suggests that PD patients with hypersexuality express sexual impulsivity and compulsivity^47^ and this was observed in the findings of our study (for example having sex with up to 15 different men in one day; increased requests for sex towards the partner).

**Fig. 2:**
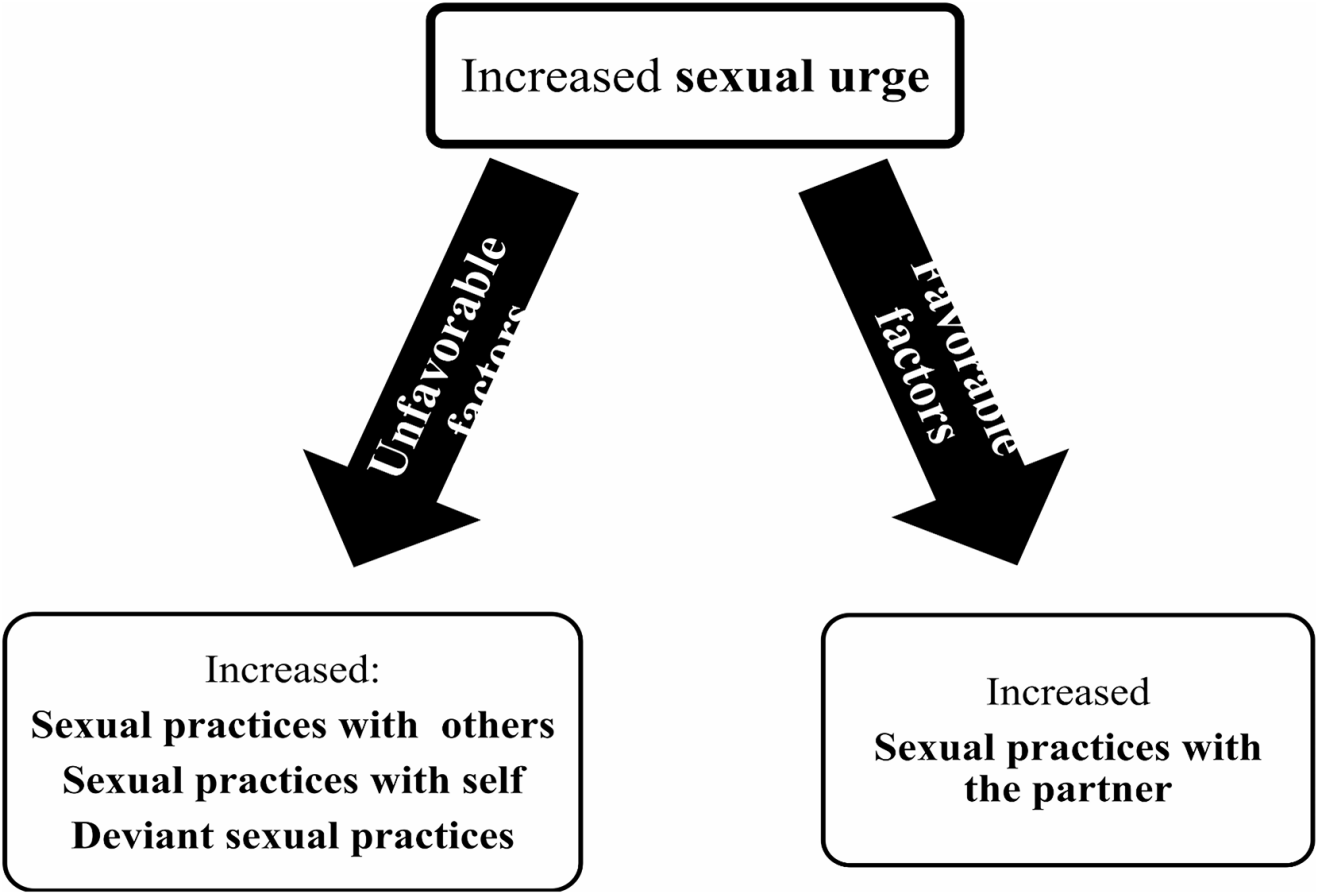
Possible connection between sexual urges and sexual practices.

In terms of sexual practices, the study found that all patients experienced increased sexual urges/desires, which led to various sexual practices. Heightened desire did not result in more frequent sexual activity with the partner, as most patients reported either unchanged or reduced frequency. This was often due to partner dissatisfaction or absence. Consequently, patients sought sexual satisfaction with themselves through masturbation or viewing explicit material, or through promiscuous encounters with sex workers or affairs. While cautious of limited sample size and the qualitative nature of the data, the research suggests a potential link between heightened urges and enacted practices, influenced by external factors. Favorable conditions with a content and accepting partner directed focus towards the partner, whilst unfavorable circumstances led to alternative outlets (self-practices, interactions with others, and unconventional behaviours). This connection is illustrated in Fig. 2. Although this relationship has not been specifically investigated in the literature on hypersexuality in neurological disorders, it has been intimated in research regarding marriage and the family. Knox and Schacht (2016) suggest that individuals who have sexually unwilling partners might be driven to seek sexual fulfilment elsewhere^44^.

In the context of female hypersexuality and its manifestations, it is crucial to differentiate between hypersexuality and Persistent Genital Arousal Disorder (PGAD), also known as Restless Genital Syndrome (ReGS). PGAD is characterized by persistent, unwanted genital arousal, often leading to extreme masturbation and unsatisfactory orgasms, which can overlap with symptoms of hypersexuality. Given the complexity of these symptoms, further inquiry is necessary to discern whether the observed female masturbation is a result of hypersexuality and sexual preoccupation or indicative of PGAD. Studies have documented cases of women with PD experiencing PGAD, characterized by similar extreme behaviors^45–47^. Acknowledging this distinction in our study is essential, as it could influence both the interpretation of our findings and the therapeutic approaches. Therefore, we recommend discussing this potential overlap and considering it a limitation of the study, as not addressing it might lead to misinterpretations of the underlying causes of the behaviors observed in our female participants.

In terms of emotional triggers, a few PD patients indicated that negative feelings, such as sadness, triggered their hypersexual episodes. Research suggests that sexually compulsive individuals “reported engaging in sexual behaviour in response to specific negative emotional states”^48^.

The study revealed that most PD patients lacked insight into their hypersexuality. However, three patients with insight recognized the behaviour as unnatural, experienced negative emotional formulations, identified negative emotional triggers, expressed a genuine desire to overcome it, and acknowledged a lack of control. Such a relationship between these variables is a novel observation as no prior studies have explored these themes in the context of hypersexuality or impulse control behaviours in neurological disorders. The research also found that hypersexuality significantly impacted various aspects of daily life, aligning with findings from Mendez and Shapira (2013)^49^. Interestingly, patients without insight also recognized the negative impact of hypersexuality, particularly on spousal relationships, causing distress to their partners. This suggests a level of situational awareness, which may influence their overall well-being. Clinicians should be attuned to this nuanced distinction when providing care and support. A reason for the lack of insight into the hypersexuality, however, could be that the impact was not severe enough to warrant a change in insight.

Most of the patients held stigma around their hypersexuality. Some referred to being older as a reason for their embarrassment about the hypersexuality. This is consistent with the literature on stigma and sexuality in old age. A study by Dominguez and Barbagallo (2016) showed that sexuality in old age is “still conditioned by biases, prejudices”, and borne from “stereotyped vision” which considers older people as “asexual” although older people do indeed have “sexual potential to express”^50^. Moreover, patients also appeared to laugh or apologise when describing sexually explicit details of their experience with hypersexuality. Apologizing and nervous laughter may be methods used to try to balance anxiety or embarrassment and mask discomfort^51^. Some patients explicitly expressed shame due to the sexual behaviour.

The results of our study raises the question how stigma might drive behaviour. The stigma around their sexual behaviours drove patients to attempt to hide their hypersexuality and resulted in a considerable degree of about being found outThis was evident not only in the interviews but even prior during the process of recruitment. Patients who declined participation in the study indicated embarrassment as their reason for choosing not to participate, which may also be directly related to fear of being stigmatised. It is also possible that the patients who initially indicated an interest in participating did not attend their scheduled appointments may have withdrawn out of fear and embarrassment of discussing this issue. This was also evident in the refusal of two patients to have their interviews recorded, despite ensured deletion and confidentiality.

While the patients were not directly queried about their professional help-seeking behaviour and associated barriers, they did express dissatisfaction with the services provided. Barriers may arise from the stigma^52^ linked to sexuality and the sensitivity surrounding the topic for both patients and healthcare professionals. Professionals may refrain from broaching the subject to avoid discomfort, lack confidence in addressing it, or be unsure about available resources. The findings underscore that patients are not receiving adequate information and assistance for their newly-developed hypersexuality. These barriers include deficient communication, understanding, education, professional neglect, and discomfort discussing sex. Although there is no specific research on professional help-seeking barriers for sex-related issues, this aligns with Hinchcliff et al. (2005)’s study on general practitioners’ challenges in addressing sexual health issues^53^. For example, barriers to discussing sexual dysfunction in multiple sclerosis included presence of family or friends, lack of knowledge about SD, and inadequate time during the consultation^54^. This study highlighted practitioners’ unfamiliarity with diverse sexual practices and concerns about appropriate language^53^. While focused on homosexuality, parallels can be drawn to hypersexuality as both deviate from conventional norms. Notably, these professional barriers exacerbate patient distress, prolonging their silent suffering.

There are several limitations due to the preliminary nature of this study. Research into sex warrants its own set of limitations including but not limited to fearful and hesitant participants^55^. Patients tend to be too ashamed to divulge information about their sexual behaviour and pursuits^56^, especially in older populations^56^. Sexuality is considered a sensitive and private topic, which is fed into by social, cultural, moral, and legal norms and restraints^55^, and may involve stigmatised and/or illegal behaviour^57^. This limits the number of individuals willing to speak of their sexuality with health professionals, as highlighted by the 24 patients who refused to participate in our study. Additionally, we lack demographic and clinical data on this group, which further limits the generalizability of our findings. However, as previously mentioned, according to qualitative research standards, a sample size of 9 can be sufficient for generating meaningful insights, especially in studies involving sensitive topics like hypersexuality in PD. This number allows for a thorough exploration of individual experiences and contributes to theory development within the constraints of qualitative research.

Nonetheless, despite these limitations, this study delved into the nuanced phenomenology of HS in these 9 patients, exploring lived experiences, triggers, and coping mechanisms. Beyond this, it uncovered the profound impact of HS on various facets of life such as quality of life, work, and personal relationships, elucidating emotional distress and societal challenges faced by patients. Moreover, qualitative studies provide a longitudinal perspective, capturing the evolving nature of HS experiences over time. This surpasses single cross-sectional analyses, enriching our understanding of the clinical, psychological, and sociocultural dimensions associated with HS in PD. This preliminary study on hypersexuality in neurological disorders suggests multiple avenues for future research. These include the development of specialized questionnaires for hypersexuality, comparative studies across different neurological disorders to unveil unique patterns, assessments of sexual behavior in Parkinson’s patients not on medication, larger-scale investigations comparing hypersexuality in neurological disorders with sex addiction in non-neurological populations, exploration of insight determinants in hypersexuality, and studies examining barriers to professional help-seeking among patients, possibly involving general practitioners and consultants for comprehensive understanding. These proposed directions aim to fill gaps in understanding, potentially leading to enhanced diagnostic tools and more effective clinical approaches.

Stigmatizing behaviours do not deter sexual activities^58^, highlighting the need for healthcare professionals to address the associated stigma. A recent study with 100 PD patients, has shown their need for sexual information and counselling^59^. Consultants, psychologists, nurses, and GPs should receive education on hypersexuality’s negative impact to effectively inform patients. Normalizing and explaining hypersexuality, along with providing reassurance, can significantly reduce the burden on patients. It is crucial for professionals to clarify that hypersexuality in neurological disorders, especially Parkinson’s, is primarily a chemical imbalance linked to neurotransmitter effects, rather than a behavioural or personality change resulting from the disease^47^. The “Open Sexual Communication” (OSEC) model, effective in discussing sexual disorders, involves four steps to identify patient concerns and suggest appropriate interventions^60^,^61^. Sensitivity is paramount when obtaining a sexual history^60^. A thorough clinical assessment is of utmost importance^62^. If professionals feel unequipped to address this issue, they should designate a team member to handle it and offer referrals^63^. Understanding the complex nature of hypersexuality is essential for providing effective assistance.

## Electronic supplementary material

Below is the link to the electronic supplementary material.

Supplementary Material 1

## Data Availability

The datasets used and/or analysed during the current study are available from the corresponding author on reasonable request.

## Footnotes

1 To try and avoid the possibility of the same patient filling it out more than once.
