# Author Correction: A qualitative study exploring the clinical phenomenology and impact of hypersexuality in patients with Parkinson’s Disease

**DOI:** 10.1038/s41598-024-83151-7

**Published:** 2024-12-20

**Authors:** Natalie Tayim, Jalesh N. Panicker, Jennifer Foley, Caroline Selai, Walaa G. El Sheikh

**Affiliations:** 1 Doha Institute for Graduate Studies, Doha, Qatar; 2 Department of Uro-Neurology, The National Hospital for Neurology and Neurosurgery, Queen Square, UCLH NHS Foundation Trust, London, UK; 3 Department of Brain Repair and Rehabilitation, UCL Queen Square Institute of Neurology, Faculty of Brain Sciences, University College London, London, UK; 4 Department of Neuropsychology, National Hospital for Neurology and Neurosurgery, Queen Square, WC1N 3BG London, UK; 5 UCL Queen Square Insitute of Neurology, WC1N 3CG London, UK; 6 Department of Clinical and Movement Neuroscience, UCL Queen Square Institute of Neurology, Faculty of Brain Sciences, University College London, London, UK; 7 Faculty of Medicine, American University of Beirut, Beirut, Lebanon

Correction to: *Scientific Reports* 10.1038/s41598-024-79966-z, published online 20 November 2024

The authors omitted a reference in the original version of this Article.

Reference 9

“Weintraub D, Mamikonyan E, Papay K, Shea JA, Xie SX, Siderowf A. Questionnaire for Impulsive-Compulsive Disorders in Parkinson’s Disease-Rating Scale. Mov Disord. 2012;27(2):242-247. doi: 10.1002/mds.24023.”

As a result, in the Introduction,

“The literature reports varying prevalence figures for HS in PD ranging between 1.9% and 22.8%^8^, partly due to the lack of a validated HS screening questionnaire and the use of the Questionnaire for Impulsive-Compulsive Disorders in Parkinson’s Disease (QUIP) as a mean to quantify HS occurrence. XX.”

now reads:

“The literature reports varying prevalence figures for HS in PD ranging between 1.9% and 22.8%^8^, partly due to the lack of a validated HS screening questionnaire and the use of the Questionnaire for Impulsive-Compulsive Disorders in Parkinson’s Disease (QUIP) as a mean to quantify HS occurrence^9^.”

As a result of this update, the References have been renumbered.

The original Article has been corrected.

