# Supplementary material for: A qualitative study exploring the clinical phenomenology and impact of hypersexuality in patients with Parkinson’s Disease
Source: Sci Rep. 2024 Nov 20;14:28697. doi: 10.1038/s41598-024-79966-z (PMC11576738; doi:10.1038/s41598-024-79966-z)
Supplement: Supplementary file 1 — Supplementary Material 1 [file 41598_2024_79966_MOESM1_ESM.pdf]

University College London Hospitals NHS
NHS Foundation Trust
The National Hospital for Neurology and Neurosurgery
Queen Square, London
WC1N 3BG

Patient Assessment Interview
Chief Investigator: Dr Jalesh Panicker
Participant Identification Number:
Date:

Patient Assessment Interview

<u>Semi-structured interview schedule</u>

Interview length: 35-60 minutes

About the interviewee (to be extracted from patient notes)

Age:

Marital Status:

Neurological disorder:

Age of onset of neurological disorder:

Date:

Time:

INTRODUCTION

Thank you for agreeing to take part in an interview for this project.

This interview will be audio recorded. The main reason for this is to have an accurate set of data on this topic. This will help researchers analyze the data as the project develops. Rest assured that you would remain completely anonymous. All data collected is confidential. No records of the interview will be kept with your name or the name of the patient on it.

The following sections include questions about increased sexual behavior that has happened since getting (insert name of neurological disorder name). This is called hypersexuality. Please remember that sexual acts involving physical harm to others or child abuse is against the law. For this reason, please do not answer any questions that show that the patient's sexual behavior has been a threat to others or that the patient has had sexual relationships with minors.

I understand how sensitive this topic is. If any questions make you uncomfortable, you are completely free not to answer. Answer what you want. Also, if any questions are not understandable, please ask and they will be explained.

Appendix 6

University College London Hospitals **NHS**

NHS Foundation Trust

The National Hospital for Neurology and Neurosurgery
Queen Square, London
WC1N 3BG

Patient Assessment Interview

Chief Investigator: Dr Jalesh Panicker

Participant Identification Number:

Date:

GENERAL

1. **Question:** Since your (insert name of neurological condition) started, have you felt that there is something unusual about your sexual behavior?

   Probe 1: Can you tell me what is unusual?

   Probe 2: Is this still the case?

   Probe 3: How does this make you feel?

2. **Question:** Do you notice that there has been an increase in your sexual behavior and thoughts since getting (insert name of neurological condition)?

   Probe 1: Is this still the case?

   Probe 2: How does this make you feel?

3. **Question:** How old were you when the hypersexuality started?

4. **Question:** Do you think the PD caused your hypersexuality?

   Probe: Why do you think so?

5. (if patient has Parkinson's disease)

   Question: What medications were you taking when the hypersexuality started?

   Probe 1: Do you think the medication has caused your hypersexuality?

   Probe 2: (if so) Which medication?

6. **Question:** Did/do you have any other impulse control disorders such as increased gambling behavior or thought, increased eating behavior or thought, or increased buying?

   Probe 1: Which ones?

   Probe 2: When did they start?

   Probe 3: How severe do you think these behaviors or thoughts were/are?

University College London Hospitals NHS
NHS Foundation Trust
The National Hospital for Neurology and Neurosurgery
Queen Square, London
WC1N 3BG

Patient Assessment Interview
Chief Investigator: Dr Jalesh Panicker
Participant Identification Number:
Date:

7. **Question:** Did/do you notice any other changes in your behavior apart from the hypersexuality?
   Probe 1: What are they?
   Probe 2: When did these changes happen?

8. **Question:** Did you have any behavioral or cognitive disorders before the PD?
   Example of behavioral disorder is obsessive-compulsive disorder.
   Example of a cognitive disorder is perception and memory disorders.
   Probe: Can you tell me what they are?

9. **Question:** Do you have any previous addictions, such as drugs or alcohol?
   Probe: What addictions?

10. **Question:** Do you think there has been a change in the frequency of sexual intercourse with your partner or otherwise?
    Probe: Can you provide more details?

SPECIFIC

11. **Question:** Did/do you choose to please yourself sexually?
    Probe 1: How often?
    Probe 2: Did/does this leave you feeling satisfied?

12. **Question:** Did/do you feel you are promiscuous?
    Probe: What made/makes you think so?

13. **Question:** Did/do you feel sexually excited by anything unusual?
    Probe 1: Can you provide examples?
    Probe 2: When did you start noticing this?

University College London Hospitals NHS
NHS Foundation Trust
The National Hospital for Neurology and Neurosurgery
Queen Square, London
WC1N 3BG

Patient Assessment Interview
Chief Investigator: Dr Jalesh Panicker
Participant Identification Number:
Date:

14. **Question:** Which of the following have you tried since experiencing hypersexuality? I will list them and you are required to just say yes or no to each.

    Internet porn?

    Pornographic novels ?

    Uncontrollable masturbation?

    Prostitution?

    Voyeurism: getting sexual satisfaction from spying on sexual objects or acts?

    Exhibitionism: the act of showing your genitals to strangers?

    Affairs?

    Anonymous sexual encounters?

    One-night stands?

    Bath houses: communal bath places?

    Massage parlors?

    Strip clubs?

    Sexual encounters with a gender not typically interested in?

    Sexual misconduct in the workplace?

    Being aggressive with sexual partner?

    Asking for sexual partner to be aggressive?

    Bestiality: sexual encounters with animals?

    Any others that I haven't listed?

15. **Question:** Do you think your hypersexuality has negatively affected your life?

    Probe: Has it affected your

    Marital life? How so?

    Family life? How so?

    Social life? How so?

    Work? How so?

    Finances? How so?

    Health? How so?

    Mood? How so?

    Sleep? How so?

The National Hospital for Neurology and Neurosurgery
Queen Square, London
WC1N 3BG

Patient Assessment Interview
Chief Investigator: Dr Jalesh Panicker
Participant Identification Number:
Date:

Self-confidence? How so?

Quality of life? How so?

16. **Question:** Did/does your hypersexuality match your personal beliefs and values?
    Probe 1: In what way?
    Probe 2: Do you believe this limited/limits your daily activities?

17. **Question:** Did/do you try to decrease your hypersexuality or stop it altogether?
    Probe 1: Were you successful?
    Probe 2: How does this make you feel?

18. **Question:** Did/do you feel more hypersexual when you had/have certain feelings such as happiness, stress, anger, or sadness?
    Probe 1: Which of these feelings?
    Probe 2: Are there any other feelings besides these?

19. **Question:** Did/do you try to hide your hypersexuality?
    Probe: How?

20. **Question:** Did/do you worry that others will find out about it?

21. **Question:** Did/do you think you are only able to feel satisfied when sex is involved?

22. **Question:** Did/do you believe you needed/need more and more risk in order to reach the same level of excitement?

23. **Question:** Did/do you feel a lot of time was/is spent on sexual behavior or thought that you would have rather used on something else?
    Probe: How much time do you think was/is spent?

University College London Hospitals NHS
NHS Foundation Trust
The National Hospital for Neurology and Neurosurgery
Queen Square, London
WC1N 3BG

Patient Assessment Interview
Chief Investigator: Dr Jalesh Panicker
Participant Identification Number:
Date:

24. **Question:** Did/do you find it hard to concentrate on other areas of your life because of thoughts and feelings about your hypersexual behavior?
    Probe: What areas?

25. **Question:** Did/do you feel that you had/have no control over your hypersexuality?

26. **Question:** What do you think will happen if the public learns about your hypersexuality?

27. **Question:** How did/does your partner feel about your hypersexuality?
    Probe 1: Have they talked about it with you?
    Probe 2: Did/does the way your partner feels affect you?
    Probe 3: How?

28. **Question:** Did/do you ever feel your partner is to blame for your hypersexuality?

29. **Question:** Have you ever felt happy with your hypersexuality?

30. **Question:** Did/do you want to overcome your hypersexuality?
    Probe: Have you made this clear to your partner?

CLOSURE

We have reached the end of our interview. I would like to thank you for being so patient. However, do you believe there is anything we have missed out that you would like to add?

Do you have any other comments about what we have discussed, or about the research as a whole?

We will send you a summary of the research findings when it becomes available.

Thank you so much for your participation.
